# Experiences of novice student team members in evidence synthesis: Study protocol for a study within a review.

**DOI:** 10.12688/hrbopenres.14154.3

**Published:** 2026-01-18

**Authors:** Sarah Dillon, Alex Gall, Elayne Ahern, Aoife Whiston, Rose Galvin

**Affiliations:** 1 School of Allied Health, University of Limerick Faculty of Education and Health Sciences, Limerick, County Limerick, V94 T9PX, Ireland; 2 Health Research Institute, University of Limerick, Limerick, County Limerick, V94 T9PX, Ireland; 3 Department of Psychology, University of Limerick Faculty of Education and Health Sciences, Limerick, County Limerick, V94 T9PX, Ireland

**Keywords:** Evidence synthesis, systematic review, novice researchers, student experiences

## Abstract

**Background:**

Evidence synthesis plays a vital role in healthcare research, informing clinical practice and policy. Increasingly, students are involved in reviews, yet their experiences, including the barriers and facilitators for participation, remain underexplored. This study aims to examine the barriers and opportunities encountered by novice student team members in evidence synthesis.

**Methods:**

This Study Within A Review (SWAR) will adopt a mixed-methods approach. Undergraduate and postgraduate students in health-related disciplines engaged in or having just completed their first evidence synthesis at the University of Limerick (Ireland) will be recruited through academic networks and word of mouth. Data will be collected primarily via focus groups, in addition to a short online survey. Thematic analysis will be employed to analyse qualitative data, while descriptive statistics will be used for survey responses. Qualitative and quantitative data will be collected concurrently and narratively integrated at the point of analysis using a weaving approach.

**Results:**

This study aims to describe the experiences of students involved in evidence synthesis, identifying barriers and facilitators related to their participation. Findings will inform recommendations for improving evidence synthesis training, mentorship, and student engagement strategies.

**Conclusion:**

Understanding student experiences in evidence synthesis may inform the development of targeted training and support mechanisms. The findings will provide valuable insights for academic institutions and research teams seeking to optimise student involvement in evidence synthesis.

## Background

Evidence synthesis is a critical research methodology in healthcare, providing a structured and transparent approach to consolidating findings across studies and identifying key knowledge gaps ^1, 2^. Evidence syntheses approaches vary, based on the research question and type of data to be synthesised, with scoping reviews, qualitative syntheses, and systematic reviews among popular approaches ^3^. Furthermore, evidence synthesis plays a central role in informing policy and practice recommendations. This is especially important in light of the association between the implementation of evidence-based decision-making and positive health outcomes ^4^. Evidence synthesis is a popular methodological approach, with rapid increases in their publication in the past decade ^5^. Although clear guidelines for the reporting and conduct of the varying evidence syntheses types exist ^6– 9^, quality is inconsistent ^10^.

One key factor influencing the quality and efficiency of evidence synthesis is the composition and expertise of the review team. Increasingly, novice researchers and students are included in evidence synthesis teams, often as part of research training or formal coursework. The involvement of students in research brings with it numerous benefits. Student participation in evidence synthesis fosters skill development in systematic searching, critical appraisal, and synthesis methodologies, all of which are foundational in developing research competence. Moreover, the involvement of students in research projects enables the development of knowledge, which is often integrated into clinical practice ^11^. However, novices may face unique barriers and facilitators during the evidence synthesis process, with more research needed to investigate what these are and how successful student participation in evidence syntheses can be nurtured.

Addressing this gap is particularly relevant in the healthcare domain, where students engaged in evidence synthesis may later apply these skills in clinical practice, policymaking, or academic research. Given that health and social care students represent an important subset of end-users of evidence synthesis, evaluating their experiences with a view to improvement may enhance research literacy and encourage more healthcare professionals to contribute to evidence syntheses. Moreover, many clinicians return to education, completing postgraduate programmes as a form of professional development. It has been suggested that establishing and fostering partnerships with policy makers and managers may facilitate the implementation of systematic review findings ^12^. Similarly, encouraging the participation of currently practicing clinicians in the conduct of reviews may enhance the relevance and impact of the output itself.

Although numerous publicly available training resources exist to support students in conducting evidence syntheses, there has been limited research on their actual experiences, barriers, and perceived facilitators during the review process. Existing literature has primarily focused on early career researchers ^13^. Yet, there is a distinct gap in understanding how students navigate the evidence synthesis process. Unlike early career researchers, students may have less access to structured mentorship, formal research networks, and methodological expertise ^14^, which can impact their ability to engage meaningfully in evidence synthesis projects. Identifying factors that could either facilitate or impede student participation in these projects may help create a more supportive environment for their involvement and enhance the quality of outputs. Furthermore, examining students' experiences can provide insights into their learning journeys, the knowledge they acquire, and whether their learning is applied to practice.

### Aims and objectives

This research adopts a Study Within A Review (SWAR) approach, which is designed to evaluate methodological processes within evidence synthesis ^15^. SWARs aim to generate evidence on how evidence syntheses are conducted, structured, and reported, thereby informing best practices. By employing this approach, the research aims to understand student experiences and training in relation to evidence synthesis. Specifically, this SWAR aims to address the following objectives:

- Explore the experiences of novice student team members who have worked on/are working on evidence synthesis projects.

Identify key barriers and facilitators influencing the ability of student researchers to contribute effectively to evidence synthesis projects. Novice student team members will be defined as students currently participating or those with recent experience (within the past year) of participating in their first evidence synthesis project.

## Methods

### Design

This SWAR will be conducted in the Faculty of Education and Health Sciences within the University of Limerick (Ireland), which spans many health-related disciplines and offers both undergraduate and postgraduate study opportunities. This SWAR will adopt a mixed method, embedded design ^16^. A qualitative dominant approach will be taken, with quantitative data collection aiming to complement qualitative findings. For the qualitative element, focus groups will explore the experiences and opinions of students in relation to evidence synthesis. A constructivist approach will be taken, acknowledging the creation of meaning through interaction ^17^. The quantitative element will consist of a short accompanying survey detailing demographic information and three Likert Scale questions pertaining to satisfaction with the evidence synthesis experience. Qualitative and quantitative elements will be collected concurrently. The topic guide and survey are available in Section 6, Extended Data. Given the primarily qualitative approach, the Consolidated Criteria for Reporting Qualitative Studies (COREQ) Checklist will be adhered to for qualitative elements ^18^. The Good Reporting of A Mixed Methods Study (GRAMMs) will also be used to ensure transparent reporting ^19^. In lieu of mixed method protocol guidelines, this protocol has been reported in accordance with established guidelines for qualitative protocol reporting ^20^.

### Research team roles and experience

This research team comprises five researchers. SD and AG will be involved in facilitating the focus groups. SD (BSc, PhD) is a Chartered Physiotherapist and an Assistant Professor within the School of Allied Health. She has experience in completing and supervising several evidence synthesis projects and has completed an Evidence Synthesis Ireland Fellowship. AG (BSc) is a student within the Department of Psychology, undertaking his MSc in psychology of global mobility, inclusion and diversity in society. AG has no previous experience completing evidence synthesis projects. RG (BSc, PhD), EA (BSc, PhD) and AW (BSc, PhD) are academic staff within the Faculty of Education and Health Sciences. Each of these authors has previously conducted and supervised numerous evidence synthesis projects.

### Reflexivity

The backgrounds, beliefs, and experiences of the researchers collecting, analysing, and interpreting the qualitative data will be acknowledged. Researchers will examine their biases through the use of reflective diaries and critical discussion of interpretations during the analysis process. This aims to strengthen the trustworthiness of the study, however, the inherent subjectivity of researchers in the creation of themes and codes is recognised. Focus groups will be conducted by researchers with no prior relationship to the participants. At this point, the researcher will verify transcribed data and participants will be pseudonymised.

### Participants and recruitment

A convenience sample of students who have just completed or are in the process of completing their first evidence synthesis within a health-related discipline will be recruited via virtual learning platform announcement and word of mouth. Students at levels 8, 9 and 10 will be included (undergraduate, postgraduate (taught), and postgraduate (by research)). Participants will be considered eligible if they are (i) over 18 years old, (ii) enrolled in higher education, (iii) have access to the internet and (iv) are currently participating, or have participated within the past year, in their first evidence synthesis project in the field of health or behavioural sciences. Students must have had experience with one or more of the following: database searching, study screening, data extraction, quality appraisal or evidence synthesis. Students will be excluded if they have completed more than one evidence synthesis and therefore are no longer considered “novice”.

### Procedure

Students wishing to participate in the study will be asked to participate in a single online focus group and survey, led by members of the research team (SD or AG). Each focus group will consist of up to 6 students, in line with previous recommendations ^21^. It is envisaged that 10–15 participants will be recruited across 3 focus groups.

Focus groups will be used to explore the experiences of students who have contributed to an evidence synthesis, using an interview guide agreed a priori through discussion among the research team and consideration of previous literature. The focus group will explore topics such as satisfaction with evidence synthesis experiences, barriers and facilitators to completion, and applicability to clinical practice. Each focus group will be audio recorded and will be allocated approximately 1 hour. One interviewer (SD/AG) will be present at each focus group and will make field notes. Groups will not be moderated by research team members who have previously worked in a supervisory capacity with focus group members. Students will also be asked to complete a short custom survey (5–10 minutes in duration) (Qualtrics), detailing their clinical position (if any), evidence synthesis experience, and perceptions of the experience. Students will be made aware that all experiences and opinions will be welcomed, including those of a negative nature, from the outset of the study in an attempt to reduce social desirability bias.

### Data analysis

Focus groups will be transcribed verbatim by the investigators and verified against audio recordings for accuracy. Data will be analysed in line with Braun and Clarke’s reflexive thematic analysis ^22, 23^. Two researchers (SD and AG) will independently familiarise themselves with the data by reading transcripts and listening to audio recordings. These researchers will independently code interviews using NVivo (Version 15), with subsequent comparison and discussion of codes, as well as the creation of themes emerging from the codes. Disagreements in codes will be discussed until a consensus has been reached; if this is not possible, a third reviewer (EA) will aid in reaching a decision. Themes will be presented, with exemplar quotations provided. Quantitative survey data will be used to complement qualitative elements and will be analysed using descriptive statistics in SPSS (IBM). Qualitative and quantitative data will be analysed independently and merged with qualitative data at the point of interpretation ^16^. Narrative integration will be employed by weaving qualitative and quantitative findings on a theme-by-theme basis ^24^.

### Data management and storage

The Qualtrics survey data will be collected anonymously. Transcriptions from the focus groups will be downloaded to the PI's password-protected GDPR-compliant University one-drive account. These transcriptions will be pseudonymised by removing participant names and replacing them with an ID within 7 days of data collection. No attempt will be made to link survey and transcript data. Data will be stored in line with the University Policy for data retention.

### Ethical considerations

Ethical approval for this study has been obtained from the Faculty of Education and Health Sciences Research Ethics Committee at the University of Limerick on 27/11/2024 (Approval Number: 2024_11_23_EHS). The information sheet explaining the study will be sent to interested participants to review. On the day of data collection, written informed consent will be collected via the Qualtrics platform. All participants will be notified of their right to withdraw at any time, up until data anonymisation. Minimal risks are anticipated for participants in this study.

### Dissemination

The findings of this study will be disseminated via journal publication and locally via a “Knowledge Exchange Event”.

### Study status

At the time of protocol submission, recruitment has commenced, with data collection on-going.

## Conclusions

This SWAR aims to address a critical gap in the literature by exploring the experiences of novice student contributors to evidence synthesis projects, with a particular focus on the barriers and facilitators encountered throughout the process. As evidence synthesis plays an increasingly vital role in healthcare research and decision-making, it is essential to understand how students, both as current learners and future professionals, engage with this methodology.

Findings from this SWAR will have implications for academic institutions and research teams seeking to involve students in evidence synthesis projects. The identification of key facilitators and barriers may be used to inform the development of tailored training resources, mentorship strategies, and support mechanisms to enhance student engagement and skill development. Furthermore, by increasing stakeholder literacy in evidence synthesis, this research may contribute to broader efforts to integrate evidence-based practice into healthcare education and clinical settings.

## Data Availability

No underlying data are associated with this study. The OSF project contains the following extended data: Open Science Framework: OSF | Experiences of novice team members in evidence synthesis: Barriers, facilitators and opportunities for the future. https://osf.io/y5eh7/ ^25^. The folder “Consent Form and Participant Information Sheet” contains the consent form and information sheet. The folder “Survey and Topic Guide” contains the survey questions and focus group topic guide. The folder “SWAR Registration” contains the published Protocol as per the SWAR Repository Store. The folder “Checklist” contains the qualitative protocol checklist ^20^. Data are available under the terms of the Creative Commons Attribution 4.0 International licence (CCBY 4.0 Attribution International Deed). The Northern Ireland Network for Trials Methodology Research. SWAR Repository Store. SWAR registration for “Experiences of novice team members in evidence synthesis: Barriers, facilitators and opportunities for the future.” SWAR44 Sarah Dillon, Elayne Ahern, Aoife Whiston, Rose Galvin (2024 SEP 03 2359).pdf Data will be reported in line with the COREQ ^18^ and GRAMM ^19^ Guidelines. This protocol has been reported in line with the ObsQual checklist in the absence of mixed method protocol reporting guidelines ^20^.
