# Supplementary material for: Experiences of novice student team members in evidence synthesis: Study protocol for a study within a review
Source: HRB Open Res. 2026 Jan 18;8:74. Originally published 2025 Jul 2. [Version 3] doi: 10.12688/hrbopenres.14154.3 (PMC12754357; doi:10.12688/hrbopenres.14154.3)
Supplement: Peer review history

# Reviewer report: Reviewer response for version 3

**DOI:** 10.21956/hrbopenres.15775.r52945

**Published:** 2026-01-24

**Authors:** Gregory Whitley, Georgia Charles, Isobel Abbott

**Affiliations:** 1 School of Health and Care Sciences, University of Lincoln, Lincoln, England, UK; 2 Clinical Audit and Research Unit, East Midlands Ambulance Service NHS Trust, Lincoln, UK; 3 East Midlands Ambulance Service NHS Trust, Lincoln, UK; 4 East Midlands Ambulance Service NHS Trust, Nottingham, Nottinghamshire, UK

Thank you for addressing our comments. The work is much improved. Nothing else to add. Well done.

Is the study design appropriate for the research question?

Yes

Is the rationale for, and objectives of, the study clearly described?

Yes

Are sufficient details of the methods provided to allow replication by others?

Partly

Are the datasets clearly presented in a useable and accessible format?

Not applicable

Reviewer Expertise:

Paramedicine, emergency medicine, evidence synthesis, mixed methods.

We confirm that we have read this submission and believe that we have an appropriate level of expertise to confirm that it is of an acceptable scientific standard.

# Reviewer report: Reviewer response for version 3

**DOI:** 10.21956/hrbopenres.15775.r52946

**Published:** 2026-01-20

**Authors:** Guilherme Henrique Dalaqua Grande

**Affiliations:** 1 University of Western São Paulo, Presidente Prudente, Brazil

The authors have satisfactorily addressed all the comments and suggestions raised by the reviewers. The revised version demonstrates careful consideration of each point, resulting in clear improvements to the quality and clarity of the manuscript.

Is the study design appropriate for the research question?

Yes

Is the rationale for, and objectives of, the study clearly described?

Yes

Are sufficient details of the methods provided to allow replication by others?

Partly

Are the datasets clearly presented in a useable and accessible format?

Not applicable

Reviewer Expertise:

musculoskeletal disorders, falls prevential and systematic review.

I confirm that I have read this submission and believe that I have an appropriate level of expertise to confirm that it is of an acceptable scientific standard.

# Reviewer report: Reviewer response for version 3

**DOI:** 10.21956/hrbopenres.15775.r52943

**Published:** 2026-01-19

**Authors:** Maura Dowling

**Affiliations:** 1 University of Galway, Galway, County Galway, Ireland

No further comments to make.

Is the study design appropriate for the research question?

Yes

Is the rationale for, and objectives of, the study clearly described?

Yes

Are sufficient details of the methods provided to allow replication by others?

Partly

Are the datasets clearly presented in a useable and accessible format?

Not applicable

Reviewer Expertise:

Evidence synthesis (Qualitative evidence synthesis, Scoping reviews); qualitative research, cancer nursing.

I confirm that I have read this submission and believe that I have an appropriate level of expertise to confirm that it is of an acceptable scientific standard.

# Reviewer report: Reviewer response for version 2

**DOI:** 10.21956/hrbopenres.15720.r51931

**Published:** 2025-12-30

**Authors:** Guilherme Henrique Dalaqua Grande

**Affiliations:** 1 University of Western São Paulo, Presidente Prudente, Brazil

The protocol is well-constructed, methodologically coherent, and appropriately aligned with its stated aims. The study design is suitable for addressing the research objectives, and ethical and operational considerations are clearly presented. But I have some concerns that the authors could improve for indexing.

**Justification for the SWAR Framework**

  Further clarification on how this study operationalises the SWAR framework would strengthen methodological justification.

**Participant Eligibility Criteria**

 Inclusion criteria lack detail. It is unclear whether the type of evidence synthesis undertaken, timing of completion, and academic discipline should influence eligibility or stratification.

**Moderation and Researcher–Participant Relationship**

 Although reflexivity is addressed, potential prior relationships between moderators and participants are not explained. This is particularly relevant for COREQ compliance.

**Bias Mitigation Strategies**

 Potential biases—such as social desirability bias, moderator influence, and heterogeneity across academic levels—are not explicitly discussed, nor are strategies for their mitigation.

**Data Management and Storage Procedures**

 Details regarding secure data storage, duration of retention, and data protection processes are not fully outlined.

**Resolution of Coding Discrepancies**

 The protocol does not specify how disagreements between independent coders will be resolved during thematic analysis.

Is the study design appropriate for the research question?

Yes

Is the rationale for, and objectives of, the study clearly described?

Yes

Are sufficient details of the methods provided to allow replication by others?

Partly

Are the datasets clearly presented in a useable and accessible format?

Not applicable

Reviewer Expertise:

musculoskeletal disorders, falls prevential and systematic review.

I confirm that I have read this submission and believe that I have an appropriate level of expertise to confirm that it is of an acceptable scientific standard, however I have significant reservations, as outlined above.

# Response

**Published:** 2026-01-06

**Authors:** Sarah Dillon

**Affiliations:** School of Allied Health, University of Limerick Faculty of Education and Health Sciences, Limerick, County Limerick, Ireland

*Many thanks to each reviewer for taking the time to review our manuscript. We appreciate your comments and agree that they have improved the quality of this protocol, which we have now updated. We will be mindful of your valuable input as we move forward with the research.*

**Reviewer 4**

*Thank you for your time in reading and reviewing our manuscript. We appreciate your comments and have responded to each of them below.*

**Justification for the SWAR Framework**

  Further clarification on how this study operationalises the SWAR framework would strengthen methodological justification.

*Thank you, we agree with this point, one that is similar to what has been stated by Reviewer 2. We have reflected on this point and have decided that the framing of the SWAR approach may not have been optimal in the text. We have changed the way we have framed this throughout.*

*Abstract:*

*“This Study Within A Review (SWAR) will use a mixed-methods approach.”*

*From aims/objectives:*

*“This research adopts a Study Within A Review (SWAR) approach,  which is designed to evaluate methodological processes within evidence synthesis ^15^ . SWARs aim to generate evidence on how evidence syntheses are conducted, structured, and reported, thereby informing best practices. By employing this approach, the research aims to understand student experiences and training in relation to evidence synthesis. Specifically, this SWAR aims to address the following objectives…”*

**Participant Eligibility Criteria**

 Inclusion criteria lack detail. It is unclear whether the type of evidence synthesis undertaken, timing of completion, and academic discipline should influence eligibility or stratification.

*Thanks for the suggestion. More detail has been added to this section to address the valid points raised regarding the lack of detail:*

*“A convenience sample of students who have just completed or are in the process of completing their first evidence synthesis within a health-related discipline will be recruited via virtual learning platform announcement and word of mouth. Students at levels 8, 9 and 10 will be included (undergraduate, postgraduate (taught), and postgraduate (by research)). Participants will be considered eligible if they are (i) over 18 years old, (ii) enrolled in higher education, (iii) have access to the internet and (iv) are currently participating, or have participated within the past year, in their first evidence synthesis project in the field of health or behavioural sciences. Students must have had experience with one or more of the following: database searching, study screening, data extraction, quality appraisal or evidence synthesis. Students will be excluded if they have completed more than one evidence synthesis and therefore are no longer considered “novice”.”*

**Moderation and Researcher–Participant Relationship**

 Although reflexivity is addressed, potential prior relationships between moderators and participants are not explained. This is particularly relevant for COREQ compliance.

*Thanks for this point, We have added additional text to address this point:*

*“Focus groups will be conducted by researchers with no prior relationship to the participants. At this point, the researcher will verify transcribed data and participants will be pseudonymised.”*

*Additionally, the following statement is in the procedure:*

*“Groups will not be moderated by research team members who have previously worked in a supervisory capacity with focus group members.”*

**Bias Mitigation Strategies**

 Potential biases—such as social desirability bias, moderator influence, and heterogeneity across academic levels—are not explicitly discussed, nor are strategies for their mitigation.

*This is a fair point, and we have edited the text to address this. The following line has been inserted:*

*“Students will be made aware that all experiences and opinions will be welcomed, including those of a negative nature, from the outset of the study in an attempt to reduce social desirability bias.”*

*We believe that we have made efforts to address the potential for moderator bias, by implementing a focus group topic guide which has been prepared a priori, though we acknowledge that the influence of the moderator cannot be completely removed. We have also addressed the potential biases brought by moderators in the following lines:*

*“Researchers will examine their biases through the use of reflective diaries and critical discussion of interpretations during the analysis process. This aims to strengthen the trustworthiness of the study, however, the inherent subjectivity of researchers in the creation of themes and codes is recognised.”*

*We acknowledge that the differing academic levels may also change results, and aim to bring this point forward to our interpretations of research, or have it reflected in the limitations.*

**Data Management and Storage Procedures**

 Details regarding secure data storage, duration of retention, and data protection processes are not fully outlined.

*Thank you for this suggestion. As section has been inserted to address the concerns raised:*

***“Data Management and Storage***

*The Qualtrics survey data will be collected anonymously. Transcriptions from the focus groups will be downloaded to the PI's password-protected GDPR-compliant University one-drive account. These transcriptions will be pseudonymised by removing participant names and replacing them with an ID within 7 days of data collection. No attempt will be made to link survey and transcript data. Data will be stored in line with the University Policy for data retention.*”

**Resolution of Coding Discrepancies**

 The protocol does not specify how disagreements between independent coders will be resolved during thematic analysis.

*This is a valid point. We have added a sentence to address this concern:*

*“Disagreements in codes will be discussed until a consensus has been reached, if this is not possible, a third reviewer (EA) will aid in reaching a decision.”*

# Reviewer report: Reviewer response for version 2

**DOI:** 10.21956/hrbopenres.15720.r51377

**Published:** 2025-12-30

**Authors:** Laura Pickell

**Affiliations:** 1 Carleton University Department of Health Sciences, Ottawa, Ontario, Canada

This review examines the published research protocol entitled “Experiences of novice student team members in evidence synthesis: Study protocol for a study within a review,” a study within a review (SWAR) exploring the experiences of students as novices conducting knowledge synthesis research.

 The rationale and aims of the study are clearly outlined and accurate. An additional relevant scoping review by Premji, Hayden, and Rutherford (2021) may also be useful in further situating this work within the existing literature. (Ref 1 - Premji Z, et al., 2021)

 The study design is appropriate in addressing the study research questions and demonstrates strong alignment between the study aims, design, and planned data collection and analysis. A notable strength is the inclusion of strategies to enhance trustworthiness and transparency, such as reflexivity, the use of agreed-upon interview guides, adherence to reporting guidelines, and researcher triangulation in the thematic analysis. The protocol also demonstrates careful attention to ethical considerations.

 The comments from the previous reviewer regarding the need to mitigate potential conflicts of interest or undue influence over students are important and are echoed here. It is also suggested that caution be applied in the data analysis and interpretation given the potential for self-report bias. Transparent reporting of the limitations associated with this form of data collection will therefore be important. In addition, maintaining detailed records as an audit trail is encouraged to further enhance transparency, particularly to document and justify any changes or additions made to focus group or survey questions over the course of the study.

Is the study design appropriate for the research question?

Yes

Is the rationale for, and objectives of, the study clearly described?

Yes

Are sufficient details of the methods provided to allow replication by others?

Yes

Are the datasets clearly presented in a useable and accessible format?

Not applicable

Reviewer Expertise:

scholarship of teaching and learning (SoTL), health education, evidence synthesis and research methods instruction

I confirm that I have read this submission and believe that I have an appropriate level of expertise to confirm that it is of an acceptable scientific standard.

## References

[1] : Teaching Knowledge Synthesis Methodologies in a Higher Education Setting: A Scoping Review of Face-to-Face Instructional Programs. *Evidence Based Library and Information Practice* .2021;16(2) : 10.18438/eblip29895 111-144 10.18438/eblip29895

# Response

**Published:** 2026-01-06

**Authors:** Sarah Dillon

**Affiliations:** School of Allied Health, University of Limerick Faculty of Education and Health Sciences, Limerick, County Limerick, Ireland

*Many thanks to each reviewer for taking the time to review our manuscript. We appreciate your comments and agree that they have improved the quality of this protocol, which we have now updated. We will be mindful of your valuable input as we move forward with the research.*

**Reviewer 3**

 The comments from the previous reviewer regarding the need to mitigate potential conflicts of interest or undue influence over students are important and are echoed here. It is also suggested that caution be applied in the data analysis and interpretation given the potential for self-report bias. Transparent reporting of the limitations associated with this form of data collection will therefore be important. In addition, maintaining detailed records as an audit trail is encouraged to further enhance transparency, particularly to document and justify any changes or additions made to focus group or survey questions over the course of the study.

*Thank you very much for these valuable comments. We acknowledge that this is indeed a limitation and we will ensure to mention the limitations of self-report bias in the final manuscript. Thank you also for your other points regarding transparency and audit tracking, we will take these on board.*

# Reviewer report: Reviewer response for version 2

**DOI:** 10.21956/hrbopenres.15720.r51379

**Published:** 2025-12-25

**Authors:** Gregory Whitley, Isobel Abbott, Georgia Charles

**Affiliations:** 1 School of Health and Care Sciences, University of Lincoln, Lincoln, England, UK; 2 Clinical Audit and Research Unit, East Midlands Ambulance Service NHS Trust, Lincoln, UK; 3 East of England Ambulance Service NHS Trust, Lincoln, UK

Thank you for the opportunity to review your revised manuscript (Version 2), titled “Experiences of novice student team members in evidence synthesis: Study protocol for a study within a review”. Along with two previous students of mine (Isobel Abbott and Georgia Charles – novice students who have conducted evidence synthesis) I have reviewed this work and concluded that overall, the work is good quality. You have responded well to Reviewer #1. I have provided some comments below you may wish to consider – I believe these will improve the quality of your manuscript.

 Major comments include:

 None.

 Minor comments include:

 There is room for improvement with the abstract in our opinion:

 Abstract – Methods – It would be helpful to explain the geographic remit of your recruitment (state that this study will be conducted within the University of Limerick, Ireland) and specify that undergraduate and postgraduate students will be recruited.

 You use the phrases “challenges and facilitators”, “barriers and opportunities” and barriers and facilitators” throughout, it would be helpful to refer to one of these consistently throughout the manuscript.

 Abstract – Methods – You have identified the study as mixed methods, however there is no description of integration techniques within the abstract – this is key to mixed methods research.

 Background - It would be helpful to define what is meant by a “novice” student team member by providing a definition of what a novice is, for the purpose of this study.

 Background - “Increasingly, novice researchers and students are included in evidence synthesis teams, either as part of research training or formal coursework.” – It may be better to say “…often as part of research training or formal coursework.” As students may engage with evidence synthesis by other means.

 Aims and Objectives - You have stated that the study is embedded with the SWAR framework – from what I can see from the reference this isn’t a “framework” as such, rather guidance. Maybe better to state you have followed SWAR guidance? I raise this because stating you used a framework makes me wonder what the framework looks like, what the steps are, etc. I would have asked you to describe the framework within this protocol.

 Methods – Design  – I’m assuming that “This SWAR will be conducted in the Faculty of Education and Health Sciences” means this is a University of Limerick only study? It would be helpful to make that clear: “This SWAR will be conducted in the Faculty of Education and Health Sciences, University of Limerick, …”

 Methods – Participants and Recruitment – While the study notes that students were recruited via a convenience sample, it would be useful to clarify the exact inclusion and exclusion criteria that were applied to ensure that only novice student participants were recruited. For example, what do you mean by “just completed” – what timeframe does this include? Were any exclusion criteria applied?

 Methods – Data analysis – The final statement alludes to data integration, however there is no description of how this will be performed. You might consider the use of a joint display, for example? https://pubmed.ncbi.nlm.nih.gov/26553895/

 Methods – Data analysis – Given the diversity of data, can you say a little about how the results will be presented? Illustrated via a thematic map? Exemplar quotations provided? Tabular format? Joint display? Other?

Is the study design appropriate for the research question?

Yes

Is the rationale for, and objectives of, the study clearly described?

Yes

Are sufficient details of the methods provided to allow replication by others?

Partly

Are the datasets clearly presented in a useable and accessible format?

Not applicable

Reviewer Expertise:

Paramedicine, emergency medicine, evidence synthesis, mixed methods.

We confirm that we have read this submission and believe that we have an appropriate level of expertise to confirm that it is of an acceptable scientific standard, however we have significant reservations, as outlined above.

# Response

**Published:** 2026-01-06

**Authors:** Sarah Dillon

**Affiliations:** School of Allied Health, University of Limerick Faculty of Education and Health Sciences, Limerick, County Limerick, Ireland

*Many thanks to each reviewer for taking the time to review our manuscript. We appreciate your comments and agree that they have improved the quality of this protocol, which we have now updated. We will be mindful of your valuable input as we move forward with the research.*

 Reviewer 2:

*Thank you for your feedback, we have responded to each of your comments below.*

 Minor comments include:

 There is room for improvement with the abstract in our opinion:

 Abstract – Methods – It would be helpful to explain the geographic remit of your recruitment (state that this study will be conducted within the University of Limerick, Ireland) and specify that undergraduate and postgraduate students will be recruited.

*This information has been inserted into the abstract section:*

*Methods This Study Within A Review (SWAR) will use a mixed-methods approach.. Undergraduate and postgraduate students in health-related disciplines engaged in or having just completed their first evidence synthesis at the University of Limerick (Ireland) will be recruited through academic networks and word of mouth.*

 You use the phrases “challenges and facilitators”, “barriers and opportunities” and barriers and facilitators” throughout, it would be helpful to refer to one of these consistently throughout the manuscript.

*We have removed “challenges” and have replaced with “barriers” throughout the protocol.*

 Abstract – Methods – You have identified the study as mixed methods, however there is no description of integration techniques within the abstract – this is key to mixed methods research.

*Thanks for pointing out this oversight. This has been addressed in the following sentence:*

*“Qualitative and quantitative data will be collected concurrently and narratively integrated at the point of analysis using a weaving approach.” We have additionally updated the analysis section of the main manuscript for clarity.*

 Background - It would be helpful to define what is meant by a “novice” student team member by providing a definition of what a novice is, for the purpose of this study.

*We have inserted this into the background:*

*“Novice student team members will be defined as students currently participating or those with recent experience (within the past year) of participating in their first evidence synthesis project.”*

 Background - “Increasingly, novice researchers and students are included in evidence synthesis teams, either as part of research training or formal coursework.” – It may be better to say “…often as part of research training or formal coursework.” As students may engage with evidence synthesis by other means.

*Thanks for the editing note, we agree and have amended the sentence as advised.*

 Aims and Objectives - You have stated that the study is embedded with the SWAR framework – from what I can see from the reference this isn’t a “framework” as such, rather guidance. Maybe better to state you have followed SWAR guidance? I raise this because stating you used a framework makes me wonder what the framework looks like, what the steps are, etc. I would have asked you to describe the framework within this protocol.

*Thank you, this is a good point and one we have considered, in the context of the previous article. On reflection, we agree that this may not be the most appropriate terminology. We have changed the way we have framed this throughout.*

*Abstract:*

*“This Study Within A Review (SWAR) will use a mixed-methods approach.”*

*From aims/objectives:*

*“This research adopts a Study Within A Review (SWAR) approach, which is designed to evaluate methodological processes within evidence synthesis ^15^ . SWARs aim to generate evidence on how evidence syntheses are conducted, structured, and reported, thereby informing best practices. By employing this approach, the research aims to understand student experiences and training in relation to evidence synthesis. Specifically, this SWAR aims to address the following objectives…”*

 Methods – Design  – I’m assuming that “This SWAR will be conducted in the Faculty of Education and Health Sciences” means this is a University of Limerick only study? It would be helpful to make that clear: “This SWAR will be conducted in the Faculty of Education and Health Sciences, University of Limerick, …”

*We have made this amendment.*

*“This SWAR will be conducted in the Faculty of Education and Health Sciences within the University of Limerick (Ireland)…*”

 Methods – Participants and Recruitment – While the study notes that students were recruited via a convenience sample, it would be useful to clarify the exact inclusion and exclusion criteria that were applied to ensure that only novice student participants were recruited. For example, what do you mean by “just completed” – what timeframe does this include? Were any exclusion criteria applied?

*Many thanks for this point. We have added further clarifications to the Participants and recruitment section:*

*“A convenience sample of students who have just completed or are in the process of completing their first evidence synthesis within a health-related discipline will be recruited via virtual learning platform announcement and word of mouth. Students at levels 8, 9 and 10 will be included (undergraduate, postgraduate (taught), and postgraduate (by research)). Participants will be considered eligible if they are (i) over 18 years old, (ii) enrolled in higher education, (iii) have access to the internet and (iv) are currently participating, or have participated within the past year, in their first evidence synthesis project in the field of health or behavioural sciences. Students must have had experience with one or more of the following: database searching, study screening, data extraction, quality appraisal or evidence synthesis. Students will be excluded if they have completed more than one evidence synthesis and therefore are no longer considered “novice”.”*

 Methods – Data analysis – The final statement alludes to data integration, however there is no description of how this will be performed. You might consider the use of a joint display, for example? https://pubmed.ncbi.nlm.nih.gov/26553895/

 Methods – Data analysis – Given the diversity of data, can you say a little about how the results will be presented? Illustrated via a thematic map? Exemplar quotations provided? Tabular format? Joint display? Other?

*We agree that this was not sufficiently clarified and thank the reviewer for their suggestions. This is a qualitatively dominant mixed-methods study and will include narrative integration (weaving of qualitative and quantitative data). We have added clarification regarding how these results will be presented/analysed:*

*“Themes will be presented, with exemplar quotations provided. Quantitative survey data will be used to complement qualitative elements and will be analysed using descriptive statistics in SPSS (IBM). Qualitative and quantitative data will be analysed independently merged with qualitative data at the point of interpretation and then integrated at the inferential stage 16 . Narrative integration will be employed by weaving qualitative and quantitative findings on a theme-by-theme basis 25.”*

# Reviewer report: Reviewer response for version 2

**DOI:** 10.21956/hrbopenres.15720.r51350

**Published:** 2025-11-21

**Authors:** Maura Dowling

**Affiliations:** 1 University of Galway, Galway, County Galway, Ireland

Thank you for addressing the queries raised—no further comments.

Is the study design appropriate for the research question?

Yes

Is the rationale for, and objectives of, the study clearly described?

Yes

Are sufficient details of the methods provided to allow replication by others?

Partly

Are the datasets clearly presented in a useable and accessible format?

Not applicable

Reviewer Expertise:

Evidence synthesis (Qualitative evidence synthesis, Scoping reviews); qualitative research, cancer nursing.

I confirm that I have read this submission and believe that I have an appropriate level of expertise to confirm that it is of an acceptable scientific standard.

# Reviewer report: Reviewer response for version 1

**DOI:** 10.21956/hrbopenres.15566.r49640

**Published:** 2025-09-16

**Authors:** Maura Dowling

**Affiliations:** 1 University of Galway, Galway, County Galway, Ireland

Thank you for the opportunity to review the protocol of a study within a review (SWAR) examining the barriers and opportunities encountered by students undertaking evidence synthesis.

 The proposed reporting guidelines are appropriate, complemented by focus group interviews and a short survey that ascertains students' experiences and perceptions.  

 The protocol would benefit from an explicit explanation of which students will be included in the recruitment. Will it be students from all levels, i.e., undergraduate, postgraduate (taught), and postgraduate (PhD)?

 In the focus groups, it is outlined that, where possible, groups will not be moderated by research team members who previously worked in a supervisory capacity with focus group members. This is wise as students’ experiences of undertaking an evidence synthesis are heavily influenced by their supervisory experience (e.g. their confidence in the supervisors’ expertise in undertaking and publishing evidence syntheses and/or assistance with screening if needed).

 The protocol uses the term ‘systematic review’ when referring to evidence synthesis. It would be helpful to clarify if this term is being used in a broader sense, also to include qualitative evidence synthesis and scoping reviews. And if so, provide citations for the reporting guidelines for these as well.

Is the study design appropriate for the research question?

Yes

Is the rationale for, and objectives of, the study clearly described?

Yes

Are sufficient details of the methods provided to allow replication by others?

Partly

Are the datasets clearly presented in a useable and accessible format?

Not applicable

Reviewer Expertise:

Evidence synthesis (Qualitative evidence synthesis, Scoping reviews); qualitative research, cancer nursing.

I confirm that I have read this submission and believe that I have an appropriate level of expertise to confirm that it is of an acceptable scientific standard, however I have significant reservations, as outlined above.

# Response

**Published:** 2025-10-31

**Authors:** Sarah Dillon

**Affiliations:** School of Allied Health, University of Limerick Faculty of Education and Health Sciences, Limerick, County Limerick, Ireland

Dear Dr Dowling,

 Many thanks for your thoughtful feedback. We have carefully considered and addressed your comments in the revised version, and we are grateful for your insights, which have helped us strengthen this protocol. Please find our detailed responses to your comments below.

 Sincere thanks.

 Best wishes,

 Sarah Dillon and the rest of the authorship team.

**Reviewer: **The protocol would benefit from an explicit explanation of which students will be included in the recruitment. Will it be students from all levels, i.e., undergraduate, postgraduate (taught), and postgraduate (PhD)?

**Authors:** Students at levels 8, 9 and 10 will be included (undergraduate, postgraduate (taught), postgraduate (by research)). This line has been inserted into the protocol. Thank you for flagging this omission.

**Reviewer:** In the focus groups, it is outlined that, where possible, groups will not be moderated by research team members who previously worked in a supervisory capacity with focus group members. This is wise as students’ experiences of undertaking an evidence synthesis are heavily influenced by their supervisory experience (e.g. their confidence in the supervisors’ expertise in undertaking and publishing evidence syntheses and/or assistance with screening if needed).

**Authors**: Many thanks for this point, we agree that this is fundamental to ensure more honest reflections of the evidence synthesis experience of students.

**Reviewer**: The protocol uses the term ‘systematic review’ when referring to evidence synthesis. It would be helpful to clarify if this term is being used in a broader sense, also to include qualitative evidence synthesis and scoping reviews. And if so, provide citations for the reporting guidelines for these as well.

**Authors:** Thank you, we agree that this was inconsistently clarified.  We have clarified this in the text by ensuring evidence synthesis is referred to, rather than systematic reviews. We have inserted references to scoping review and qualitative evidence synthesis guidelines in the introduction. We have also inserted the line:

 “Evidence syntheses approaches vary, based on the research question and type of data to be synthesised, with scoping reviews, qualitative syntheses, and systematic reviews among popular approaches (3).”
